# Optical Coherence Tomography and Its Potential Utility in Below-the-Knee Intervention: Can “Seeing More” Outweigh Practical Technology Limitations?

**DOI:** 10.1016/j.jscai.2026.105580

**Published:** 2026-08-17

**Authors:** Helena Smith, Eric A. Secemsky

**Affiliations:** a Richard A. and Susan F. Smith Center for Outcomes Research, Beth Israel Deaconess Medical Center, Harvard Medical School, Boston, Massachusetts; b Division of Cardiovascular Medicine, Beth Israel Deaconess Medical Center, Harvard Medical School, Boston, Massachusetts

**Keywords:** below-the-knee intervention, endovascular therapy, intravascular ultrasound, optical coherence tomography, peripheral arterial disease

Endovascular therapy for peripheral artery disease has seen an emergence of novel treatment options, yet procedural decision making has changed surprisingly little. Specialty balloons, atherectomy devices, intravascular lithotripsy, drug-eluting technologies, and bioresorbable platforms have expanded the treatment armamentarium, but selecting between options depends largely on angiography alone, operator preference, and local practice. This disconnect is most apparent in below-the-knee (BTK) intervention, where lesions are anatomically complex, and procedural failure carries a high risk of limb loss. As BTK intervention becomes increasingly device specific, understanding lesion biology—not simply the vessel lumen—will become essential to selecting the right device strategy. In this issue of *JSCAI*, Li et al^1^ take an important step toward exploring this question.

Coronary intervention has already undergone a revolution in imaging strategy. Optical coherence tomography (OCT), along with high-definition intravascular ultrasound (IVUS), has shifted procedural planning from a superficial angiographic assessment of lesion severity to a more comprehensive evaluation of plaque composition, calcification, and precise vessel sizing.^2^ Peripheral intervention has lagged behind this evolution, owing to the length of the peripheral vasculature, complexity of vascular disease, large size differences across arterial segments, and limited prospective evidence supporting routine use. However, limited prospective studies, observational studies, and consensus documents strongly support the emerging role of intravascular imaging to guide peripheral intervention.^3^, ^4^, ^5^, ^6^, ^7^

The majority of contemporary literature supporting intravascular imaging-guided peripheral revascularization currently involves IVUS platforms. Differing from IVUS, OCT is a unique technology that provides superior near-sided resolution, offering an improved ability to quantify calcification, identify the layers of the vessel wall, and grade dissections. Li et al^1^ prospectively evaluated and confirmed the technical feasibility of OCT for BTK intervention in a cohort of 44 patients with advanced chronic limb-threatening ischemia (92.5%, Rutherford class V-VI; 47.5%, chronic total occlusions). The study established practical imaging protocols, with undiluted contrast significantly improving image quality (a clear image length increases from 54.7 to 70.2 mm; *P* = .03). Additionally, OCT demonstrated calcification in 64.5% of lesions and was able to distinguish calcium thickness, a level of tissue characterization that was undetectable with IVUS or angiography. The message is clear: if different devices are designed to treat different lesion substrates (eg, medial vs intimal calcification), those substrates must first be identified, and OCT offers superior visualization.

One of the primary take-home messages from this analysis is the detection of calcific vascular disease. Calcium is not simply a marker of disease severity, but it also determines how a lesion mechanically responds to endovascular therapy. Vessel compliance, balloon expansion, acute luminal gain, drug penetration, and, ultimately, procedural durability is influenced by the extent and distribution of calcification. Angiography frequently underestimates calcification, while IVUS, despite its strengths, is limited in its ability to quantify calcium thickness because of acoustic shadowing.^8^ OCT overcomes many of these limitations by providing high-resolution visualization of calcium arc, thickness, and longitudinal distribution. In coronary intervention, these features have become central to identifying lesions that require plaque modification before definitive treatment. There is little reason to believe that the same biological principles do not apply to tibial arteries, where medial calcification is common, inadequate lesion preparation remains a major contributor to treatment failure, and specific device types such as intravascular lithotripsy can address this lesion subtype.

Equally compelling is the observation that satisfactory angiographic results frequently conceal persistent luminal narrowing observed on OCT, despite the use of appropriately sized balloons. Although exploratory, this finding exposes a familiar weakness of angiography,^9^ where an acceptable angiographic appearance does not necessarily indicate adequate lesion modification. Significant delayed recoil is becoming increasingly recognized as a failure mechanism in BTK disease and deserves preplanned evaluation and additional treatment when recognized. Plaque fracture, residual constraining calcium, and persistent stent underexpansion may also go unrecognized. These mechanisms are clinically important because they are not only potentially correctable but also associated with poor short-term and long-term outcomes. Furthermore, if lesion preparation is inadequate, definitive therapies, such as drug delivery, may never achieve their intended effect.

Importantly, this study should not be interpreted as evidence that OCT-guided intervention improves clinical outcomes. It was appropriately designed as a feasibility study and was neither powered nor intended to demonstrate longitudinal reductions in restenosis, reintervention, amputation, or mortality. Similarly, the associations between OCT-derived calcium characteristics and adverse limb outcomes should be viewed as hypothesis generating rather than practice changing. The next challenge is no longer determining whether OCT can image tibial arteries but determining whether the additional information it provides can meaningfully alter procedural strategy and improve patient-centered outcomes, such as wound healing, freedom from reintervention, limb preservation, and survival.

Practical considerations also remain. OCT requires blood clearance for visualization, increases procedural complexity, and demands expertise in image acquisition and interpretation. Although Li et al^1^ demonstrate that OCT can be performed using acceptable contrast volumes, these factors remain relevant in patients with multilevel disease or impaired renal function. Additionally, OCT presently does not have a role in larger vessel diseases, such as aortoiliac disease, where blood cannot be sufficiently cleared. OCT should also not be viewed as a replacement for IVUS. IVUS remains advantageous for vessel sizing, identifying extravascular structures, and assessing overall vessel architecture, particularly in larger peripheral arteries, whereas OCT excels at defining superficial plaque morphology and calcium microarchitecture.^10^ Rather than competing technologies, they are complementary tools whose value depends on the clinical question being asked.

The broader implication of this study extends beyond imaging. As BTK intervention evolves from plain old balloon angioplasty toward drug-coated devices and resorbable scaffolds, the limitations of angiography become more difficult to ignore. Device innovation has outpaced angiography and stubborn beliefs that 2-dimensional imaging is sufficient to optimize BTK procedural outcomes. Li et al^1^ suggest that closing this gap may require moving beyond catheter angiography toward a more detailed understanding of lesion morphology and procedural mechanics. Whether OCT ultimately changes outcomes is yet to be proven. What this study makes increasingly difficult to defend, however, is the assumption that angiography alone provides enough information to guide contemporary BTK intervention.

## CRediT authorship contribution statement

**Helena Smith:** Writing – original draft, Writing – review & editing. **Eric A. Secemsky:** Writing – review & editing.

## Declaration of competing interest

Helena Smith declared no potential conflicts of interest with respect to the research, authorship, and/or publication of this article. Eric Secemsky receives institutional grants to Beth Israel Deaconess Medical Center from Abbott, Boston Scientific, CathWorks, Medtronic, Philips, and Shockwave Medical serves as a consultant for Abbott, Asahi, BD, Boston Scientific, Conavi, Concept Medical, Cook, Cordis, Endovascular Engineering, Evident Vascular, GE, Gore, InfraRedx, Medtronic, Penumbra, Philips, RapidAI, Rampart IC, R3, Regeneron, Shockwave Medical, Siemens, SoniVie, Teleflex, Terumo, Thrombolex, VentureMed, Verve, and Zoll.
